# Spatial, temporal, and spatiotemporal analysis of under-five diarrhea in Southern Ethiopia

**DOI:** 10.1186/s41182-018-0101-1

**Published:** 2018-06-04

**Authors:** Hunachew Beyene, Wakgari Deressa, Abera Kumie, Delia Grace

**Affiliations:** 1 College of Health Sciences, Hawassa University, P.O. Box 1560, Hawassa, Ethiopia; 2 School of Public Health, Addis Ababa University, P.O. Box 1176, Addis Ababa, Ethiopia; 3 International Livestock Research Institute, Box 30709, Nairobi, Kenya

**Keywords:** Southern Ethiopia, Under-five diarrhea, SatScan, Cluster, Spatial, Temporal

## Abstract

**Background:**

Despite improvements in prevention efforts, childhood diarrhea remains a public health concern. However, there may be substantial variation influenced by place, time, and season. Description of diarrheal clusters in time and space and understanding seasonal patterns can improve surveillance and management. The present study investigated the spatial and seasonal distribution and purely spatial, purely temporal, and space-time clusters of childhood diarrhea in Southern Ethiopia.

**Methods:**

The study was a retrospective analysis of data from the Health Management Information System (HMIS) under-five diarrheal morbidity reports from July 2011 to June 2017 in Sidama Zone. Annual diarrhea incidence at district level was calculated. Incidence rate calculation and seasonal trend analysis were performed. The Kulldorff SaTScan software with a discrete Poisson model was used to identify statistically significant special, temporal, and space-time diarrhea clusters. ArcGIS 10.1 was used to plot the maps.

**Results:**

A total of 202,406 under-five diarrheal cases with an annual case of 5822 per 100,000 under-five population were reported. An increasing trend of diarrhea incidence was observed over the 6 years with seasonal variation picking between February and May. The highest incidence rate (135.8/1000) was observed in the year 2016/17 in Boricha district. One statistically significant most likely spatial cluster (Boricha district) and six secondary clusters (Malga, Hulla, Aleta Wondo, Shebedino, Loka Abaya, Dale, and Wondogenet) were identified. One statistically significant temporal cluster (LLR = 2109.93, *p* < 0.001) during December 2013 to May 2015 was observed in all districts. Statistically significant spatiotemporal primary hotspot was observed in December 2012 to January 2015 in Malga district with a likelihood ratio of 1214.67 and a relative risk of 2.03. First, second, third, and fourth secondary hotspots occurred from January 2012 to May 2012 in Loka Abaya, December 2011 in Bursa, from March to April 2014 in Gorchie, and March 2012 in Wonsho districts.

**Conclusion:**

Childhood diarrhea was not distributed randomly over space and time and showed an overall increasing trend of seasonal variation peaking between February and May. The health department and other stakeholders at various levels need to plan targeted interventional activities at hotspot seasons and areas to reduce morbidity and mortality.

**Electronic supplementary material:**

The online version of this article (10.1186/s41182-018-0101-1) contains supplementary material, which is available to authorized users.

## Background

Globally, diarrheal disease is the second leading cause of death in children under 5 years of age, and there are nearly 1.7 billion cases of childhood diarrheal disease every year [1]. In 2015, diarrhea was a leading cause of disability-adjusted life years (DALYs) on young children [2]. If not properly treated, diarrhea will be responsible for dehydration and death in children [2–4]. Despite a decrease in the proportions of diarrheal morbidity among under-five children, a growing trend of inequalities among neighborhoods and villages of countries has been observed [2, 5, 6].

Based on the 2016 Ethiopian Demographic and Health Survey (DHS) report, access to improved water supply and sanitation facilities have shown improvements. The percentage of children aged between 12 and 23 months, who received all basic vaccinations, also increased from 14% in 2000 to 20% in 2005, 24% in 2011, and 39% in 2016. In addition, the 2 weeks under-five diarrheal morbidity prevalence reduced from 24% in 2000 to 18% in 2005, 13% in 2011, and 12% in 2016 [7]. Studies which were conducted in different times and places in Ethiopia, however, indicated that diarrhea remains a public health problem with morbidity prevalence ranging from 18 to 30.5% [8–12]. In the study area, high rate of reversion to open defecation was reported [13].

Several studies indicated that morbidity patterns of childhood diarrhea showed spatial variation, with the occurrence of clusters [14–16]. However, studies indicated that under-five diarrhea disease did not differ significantly across different study locations [17, 18]. Seasonal patterns of childhood diarrheal morbidity have been reported from different countries with different seasonal features [14, 18–21]. Diarrheal morbidity has also been observed to be influenced by metrological parameters such as precipitation and temperature anomalies in different parts of the world including Ethiopia [14, 22–25].

Analysis of disease trends in space and time provides context which can be linked to possible risk factors in a research environment [26]. Scan statistics has been used widely in the field of epidemiology for investigation of spatial, temporal, and space-time clusters of infectious disease such as hemorrhagic fever [27], *Clostridium difficile* infection clusters [28], healthcare-associated infections or colonizations with *Pseudomonas aeruginosa* [29], visceral leishmaniasis [30], typhoid fever [31], cholera [32], malaria [33], and diarrhea [14, 15].

Description of diarrheal clusters in time and space and understanding seasonal patterns is important for informed decision-making at various levels of the health department and may lead to improvements in disease surveillance. However, studies in Ethiopia which assessed the seasonal trend, spatial, temporal, and space-time clusters of diarrhea are lacking. The very few available studies used different data sources and did not include the current study area. Acute watery diarrhea (AWD) has been a public health threat since 2006 in the study area and caused the morbidity of thousands of people. These outbreaks were linked to lack of basic sanitation and safe water supply, as well as to the high sensitivity of the pathogens to variations in climatic variability [34]. In addition to this, the effect of climate change is reported in parts of the study area [35]. The present study, therefore, was conducted to investigate the seasonal distribution, purely spatial, purely temporal, and space-time clusters of childhood diarrhea in Sidama Zone, Southern Ethiopia. The study adds to the already existing knowledge, and identifying the risk areas would help in designing effective intervention mechanisms to reduce childhood diarrhea in these areas.

## Methods

### Description of the study area

**Fig. 1:**
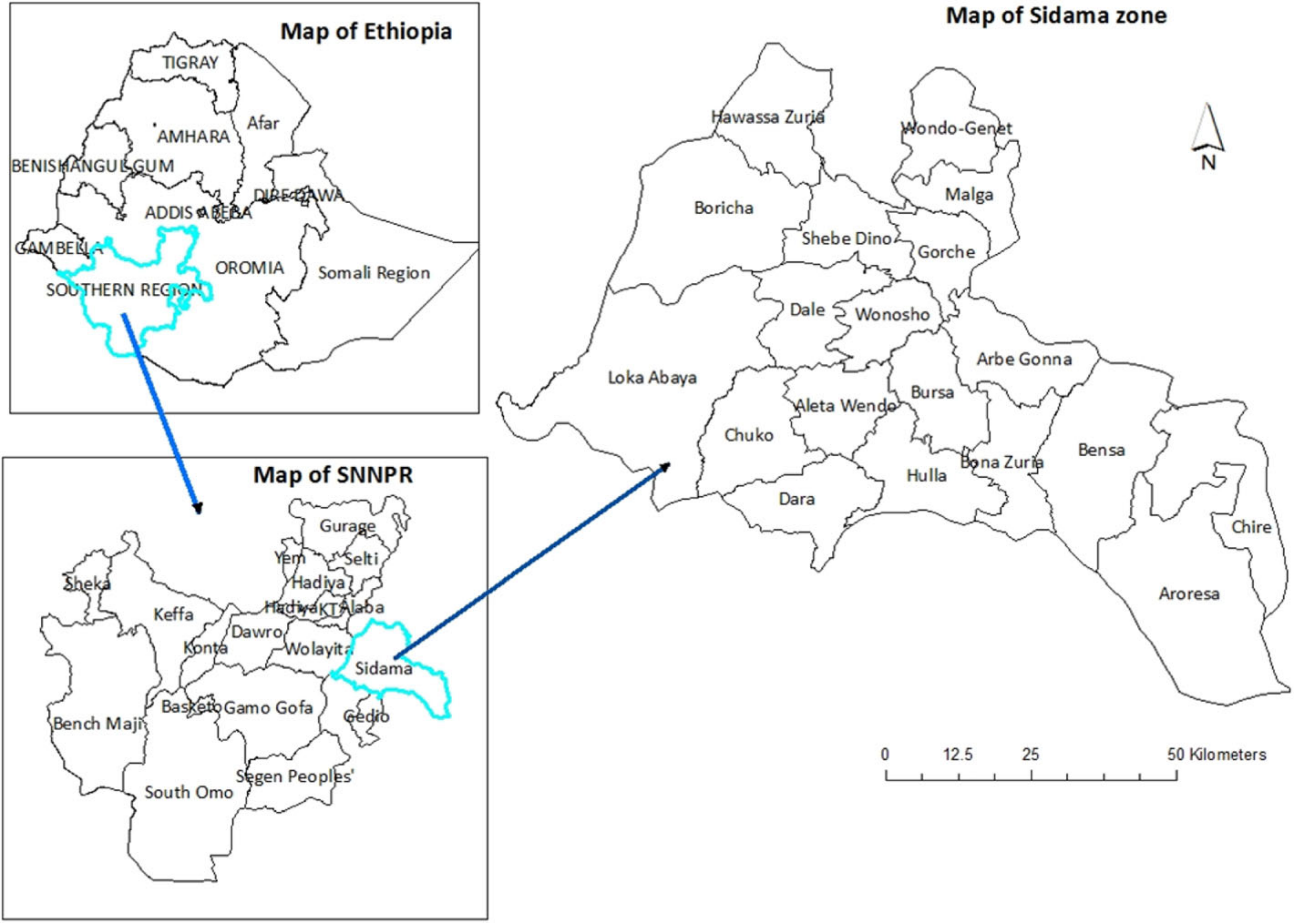
Map of the study area, Sidama Zone, Southern Ethiopia, 2017

The study was conducted in Sidama Administration Zone, Southern Ethiopia (Fig. 1). It consists of 19 rural districts and two administrative towns.

Its geographic location lies between 6°14′ and 7°18′ North latitude and 37°92′ and 39°14′ East longitude. The total area of the Sidama Administrative Zone is about 6981.8 km^2^. The administrative zone is bounded by Oromiya in North, East, and South East, with Gedieo Zone in the South and Wolayta Zone in the West. The altitude ranges between the highest peak of Garamba mountains 3500 m above sea level (masl) to low lands (1190 m) around Bilate River in Loka-Abaya and Borcha districts. The climatic condition can be described as wet moist highland (27.7%), wet moist midland (45.4%), dry midland (14.5%), dry lowland (8.6%), and wet moist lowland (3.8%) [36]. In 2017, the administrative zone had a total population of 3,668,304 with 1,849,128 male and 1,819,176 female [37].

### Study design and population

This is a retrospective longitudinal study design using the Sidama Zone HMIS under-five diarrheal morbidity report from July 2011 to June 2017. In the study area, the annual morbidity report is compiled based on the Ethiopian fiscal year which spans from July to June. The study population was all under-five children of the 19 districts who lived during the study period. Each district was represented by a geographical point location by the geographic coordinates taken from a representative location in the district. Coordinates were specified using the standard Cartesian coordinate system.

Population projection for the years 2011 to 2017 was made based on the 2007 census report as a referee population, using the population projection formula: *P* = *Po*(1 + *r*)^*t*^, where *P* is the projected total population, *Po* the reference population, *r* the regional annual population growth rate (2.9), and *t* the time of the year when the projection was made. The under-five population was calculated to be 15.6% of the total population in Southern Nations, Nationalities, and Peoples’ Region (SNNPR) [38]. The projected under-five population data of each district was specified continuously over each month for the 6 years from July 2011 to June 2017 and matched with its location ID, monthly under-five diarrheal morbidity data, and *XY* coordinate of each study location.

Cases were defined as the number of under-five children who were diagnosed to have diarrhea in each health facility of the study districts. Since July 2011, the SNNPR Health Bureau has adopted electronic Health Management Information System (HMIS), where diarrheal cases have been compiled electronically at health facility level. The compiled data are reported monthly to the Zonal Health Department and Regional Health Bureau. For this study, a 6-year monthly under-five diarrheal morbidity data from July 2011 to June 2017 was collected from the e-HMIS database. The data was collected by using a checklist. Data were collected by trained health professionals who had knowledge of the HMIS data management.

### Data analysis

The 30 years (1983–1984) average monthly rainfall, the maximum temperature, and the minimum temperature of the administrative zone were calculated from the National Meteorology Agency (NMA) Climate Analysis and Application (map room) [39]. The annual under-five diarrhea incidence per 1000 individuals in each district for the years between July 2011 and June 2017 and seasonal trend were calculated using Excel^®^. Smoothing of the data was done by calculating the 12 months (1 year) moving average followed by calculating the centered moving average (CMA). The trend of the monthly morbidity data of the 6 years was calculated by using the deseasonalized data and time. The incidence rate per 1000 under-five children, the CMA, and the trend component of the data were plotted to observe seasonal variations and trend of childhood diarrhea in the study areas (Additional file 1).

The excess hazard (the ratio of observed to expected cases greater than one) for each district was calculated by dividing the observed cases by the expected cases and plotted using the geographic information system (GIS). The expected number of cases in each area under the null hypothesis was calculated using the following formula: E[c] = p*C/P, where *c* is the observed number of cases and *p* the population in the location of interest, while *C* and *P* are the total number of cases and population respectively [40].

#### Cluster analysis

The Kulldorff scan statistic, implemented in SaTScan software (SaTSCan v9.4.4), was used to detect if diarrhea was randomly distributed over space, time, or space and time and to evaluate the statistical significance of disease clusters [41]. SaTScan was preferred among software programs capable of space-time disease surveillance analysis, as it was found to be the best-equipped package for use in surveillance system [26].

The scan statistic technique detects and evaluates the statistical significance of spatial or space-time clusters that cannot be explained by the assumption of spatial or space-time randomness, noting the number of observed and expected observations inside the window at each location. In the SaTScan software, the scanning window is an interval (in time), a circle or an ellipse (in space), or a cylinder with a circular or elliptic base (in space-time). The discrete Poisson-based model was used assuming that the reported monthly diarrheal cases are Poisson-distributed in the study area with the projected under-five underlying population at risk. The statistical principles behind the spatial and space-time scan statistics used in the SaTScan software for our specific analysis have been described in detail by Martin Kulldorff [42].

#### Purely spatial clusters

In this study, the maximum spatial cluster size of the population at risk was set to 50%. The observed diarrheal cases were compared with expected cases inside and outside of each window, and the risk ratios were estimates on the basis of Poisson distribution. The null hypothesis of the spatial scan statistic states that childhood diarrhea is randomly distributed throughout the districts of the administrative zone and that the expected event count is proportional to the population at risk. For any circular window, if the null hypothesis is statistically rejected, then the geographic area defined by the scan window can be considered as a spatial cluster. For each circle, rejection of the null hypothesis is based on a likelihood ratio statistic. The *p* value was calculated through Monte Carlo hypothesis testing, by comparing the rank of the maximum likelihood from the real data set with the maximum likelihoods from random data sets. To evaluate the statistical significance of the primary cluster, the 999 random replications of the data set are generated under the null hypothesis.

For each location and size of the scanning window, SaTScan uses a Monte Carlo simulation to test the null hypothesis, that is, there is no an elevated risk within the window as compared to outside. Under the Poisson assumption, the likelihood function for a specific window is proportional to:

$$ {\left[\frac{c}{E(c)}\right]}^c{\left[\frac{C-c}{C-E(c)}\right]}^{C-c}I\left(\right) $$

where *C* is the total number of cases, *c* is the observed number of cases within the window and *E*[*c*] is the covariate-adjusted expected number of cases within the window under the null-hypothesis, *C*-*E*(*c*) is the expected number of cases outside the window, and *I*() is an indicator function. In this study, since SaTScan is set to scan only for clusters with high rates, *I*() is equal to 1 when the window has more cases than expected under the null hypothesis, and 0 otherwise.

#### Purely temporal cluster

A purely temporal cluster analysis scanning was performed to detect the temporal clusters of childhood diarrheal cases with high rates, representing the whole geographic area but a 1-month time aggregation length. The maximum time was specified to be the default 50% within the study period. To identify clusters, a likelihood function was maximized across all locations and times. The maximum likelihood indicates the cluster least likely to have occurred by chance (primary cluster). The *p* value is obtained through Monte Carlo hypothesis testing. Secondary clusters are those that are in rank order after primary cluster by their likelihood ratio test statistic.

#### Space-time clusters

The space-time scan statistic was defined by a cylindrical window with a circular (or elliptic) geographic base and with a height corresponding to time. The base is defined exactly as for the purely spatial scan statistic, while the height reflects the time period of potential clusters. The cylindrical window is then moved in space and time so that for each possible geographical location and size, it also visits each possible time period, where each cylinder reflects a possible cluster. A likelihood ratio was calculated for each space-time window to indicate to what extent the rate of cases inside the area is higher than expected. Monte Carlo hypothesis testing is then used to indicate the significance level of specific space-time windows.

## Results

Monthly diarrheal morbidity data were collected from all the 19 study districts from the 6 years HMIS data. There was no missing data from each district during the study period. A total of 202,406 under-five diarrheal cases were reported, with an annual childhood diarrheal case of 5822 per 100,000 under-five population. The incidence rate varies from place to place and year to year, and an overall increasing trend of childhood diarrheal with seasonal variation peaking between February and May was observed. The highest incidence rate (135.8/1000) was observed in Boricha district in the year 2016/17, and the lowest incidence rate (17.3 per 1000 under-five children) was observed in Bensa district in 2016/17 and Chirie district in 2015/16 (Table 1).

**Table 1:** Yearly diarrheal incidence rate under-five children of each district, Sidama Zone, Southern Ethiopia, 2017

SN	District name	Yearly incidence rate per 1000 under-five population					
		2011/12	2012/13	2013/14	2014/15	2015/16	2016/17
1	Aleta Chuko	56.0	44.9	57.9	56.6	61.2	54.7
2	Aleta Wondo	56.0	64.0	84.7	95.4	60.2	70.6
3	Arbegona	52.4	42.5	63.9	68.3	43.9	52.0
4	Aroresa	31.6	35.5	43.8	27.7	22.4	31.2
5	Bensa	24.5	18.3	26.1	27.9	17.5	17.3
6	Bona	26.1	49.1	51.2	48.1	40.2	65.2
7	Boricha	88.3	62.0	85.1	121.8	95.7	135.8
8	Bursa	50.7	31.4	33.9	26.7	24.2	17.5
9	Chirie	18.6	91.3	82.7	37.4	17.3	27.2
10	Dale	115.2	38.5	50.7	59.6	53.4	58.1
11	Dara	59.7	48.0	53.0	64.2	52.8	44.4
12	Gorchie	45.9	42.0	65.8	63.8	46.3	32.1
13	Hawassa Zuria	40.2	37.8	30.3	59.8	41.2	51.3
14	Hulla	35.9	39.9	94.4	129.2	111.1	107.1
15	Loka Abaya	94.7	61.2	55.8	64.9	40.3	63.7
16	Malga	71.4	99.2	115.7	106.7	76.3	85.9
17	Shebedino	55.3	60.9	77.3	90.6	62.6	79.3
18	Wonsho	44.2	20.8	55.6	52.9	50.1	60.9
19	Wondogenet	32.0	40.9	56.1	63.8	79.9	75.8
Average		52.6	48.9	62.3	66.6	52.5	58.6

**Fig. 2:**
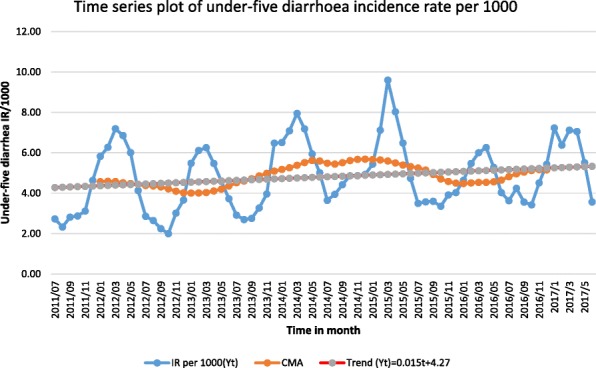
Trend and seasonal variation of under-five diarrhea rate in Southern Ethiopia, between July 2011 and June 2017

The 6 years seasonal trend of the smoothed and deseasonalized incidence rate of childhood diarrhea showed an increasing trend, with an equation of *Yt* = 0.015*t* + 4.27, which starts to increase in January and reaches its peak in February. The incidence rate starts to slowly decline through time to reach its lowest peaks in the months of July to November (Fig. 2).

**Fig. 3:**
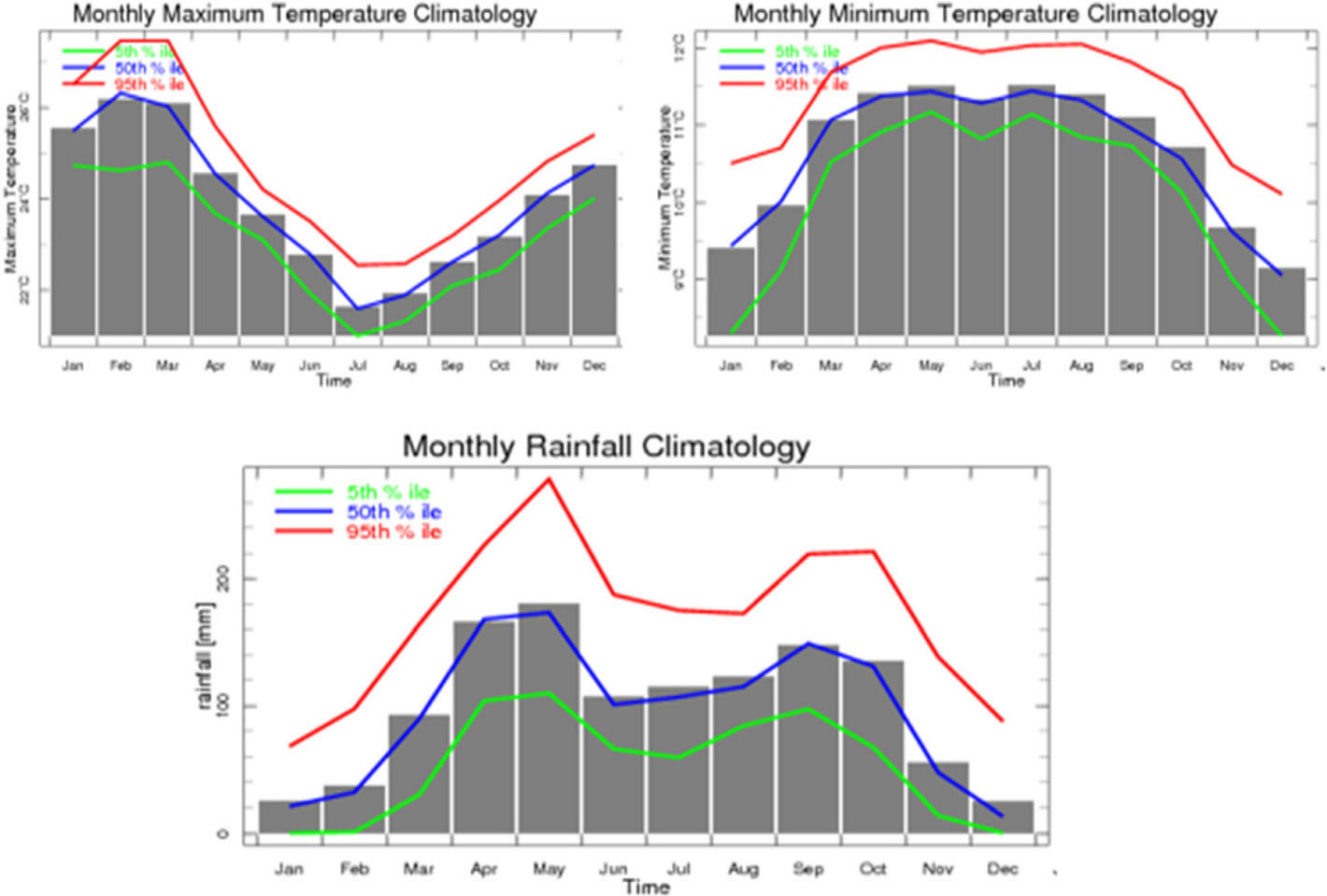
Thirty years historical (1983–2014) monthly data of precipitation, maximum temperature, and minimum temperature calculated from the National Meteorology Agency (NMA) Climate Analysis and Application (map room)

The 30 years average monthly precipitation, maximum temperature, and minimum temperature of the study area are indicated in Fig. 3. The minimum average monthly rainfall was registered in the months of November, December, January, and February, between 20 and 60 mm. The precipitation starts to increase in March (nearly 95 mm) and reaches its peak in the months of April and May (170–190 mm). A slight reduction is observed in the months of July to August (100–120 mm), then increase in September (145 mm) and October (140 mm). A significant reduction of precipitation was observed in November (nearly 60 mm) (Fig. 3). The 30 years monthly average maximum temperature of the study area showed that November, December, January, February, and March had a higher temperature, where its peak reaches in the months of February and March. It starts to drop in April and reaches its lowest in July and August and then starts to slowly increase again.

**Fig. 4:**
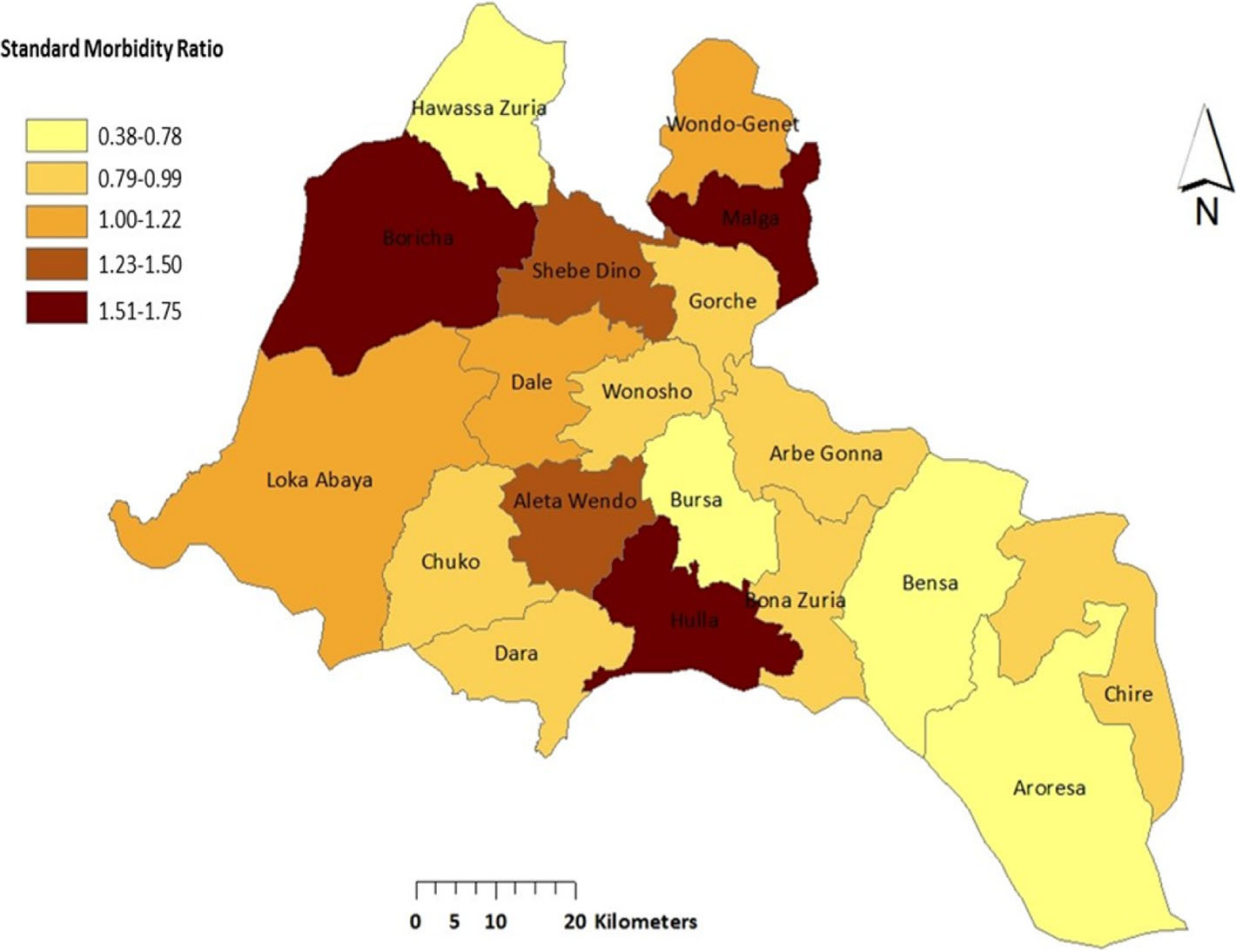
Excess risk map of under-five diarrhea from July 2011 to June 2017 in Southern Ethiopia. The areas with darker brown indicated areas with higher excess risk, and the lighter the color the lesser the excess risk of the districts on the map

The distribution of excess risk, which was defined as the ratio of the number of observed over the number of expected cases was indicated in Table 2 and Fig. 4. Eight districts (Boricha, Malga, Hulla, Aleta Wondo, Shebedion, Dale, Loka Abaya, and Wondogenet) had standard mortality ration (SMR) greater than one.

**Table 2:** Values of excess risk and relative risk of diarrhea, July 2011 to June 2017, Southern Ethiopia, 2017

SN	District	Obs.*	Exp^¥^	Obs./exp.	RR
1	Boricha	29,153	17,146.87	1.70	1.82
2	Malga	11,952	7522.60	1.59	1.63
3	Hulla	13,335	8856.59	1.51	1.54
4	Aleta Wondo	16,005	12,947.92	1.24	1.26
5	Shebedino	19,636	16,027.43	1.23	1.25
6	Loka Abaya	7351	6799.06	1.08	1.08
7	Dale	17,713	16,626	1.07	1.07
8	Wondogenet	10,792	10,668.99	1.01	1.01
9	Aleta Chuko	10,883	11,462.75	0.95	0.95
10	Arbegona	8606	9308.74	0.92	0.92
11	Dara	9786	10,638.18	0.92	0.92
12	Gorchie	6102	7226.53	0.84	0.84
13	Wensho	5045	6143.31	0.82	0.82
14	Bona	6713	8306.62	0.81	0.80
15	Chirie	6407	8252.70	0.78	0.77
16	Hawassa Zuria	6391	8528.34	0.75	0.74
17	Aroresa	6393	11,664.18	0.55	0.53
18	Bursa	3691	7100.39	0.52	0.51
19	Bensa	6452	17,178.83	0.38	0.36

*RR* relative risk, *SN* serial number

*Number of observed cases in a cluster

^¥^Number of expected cases in a cluster

### Purely spatial cluster

**Fig. 5:**
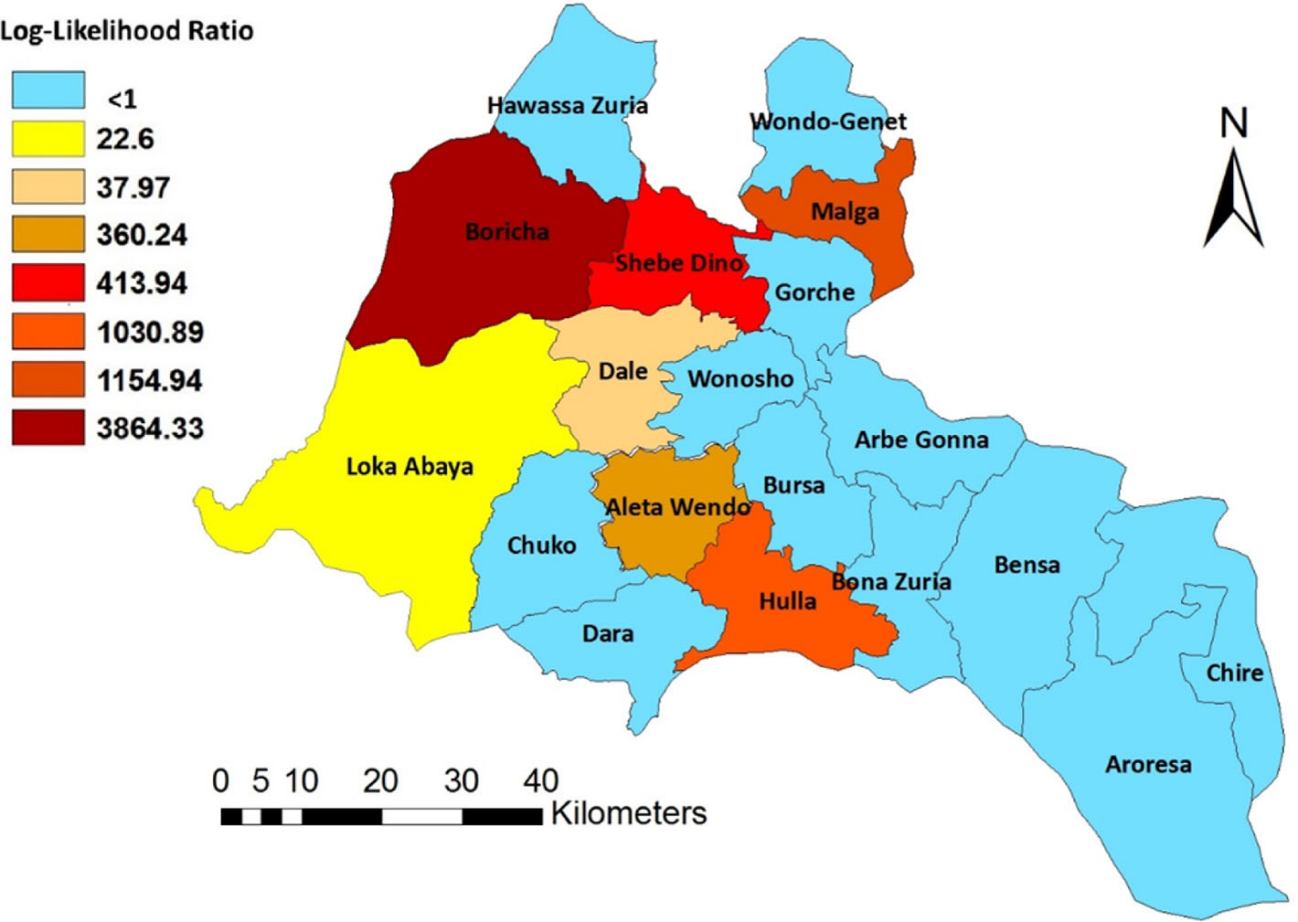
Most likely spatial cluster and secondary clusters of under-five diarrhea in Southern Ethiopia between July 2011 and June 2017. The primary cluster is found in Boricha district, and the secondary clusters were identified in Malga, Hulla, Shebedino, Aleta Wondo, Dale, and Loka Abaya districts. The order of the names of the districts is based on their likelihood ratio with decreasing order. Numerical identification of the clusters are in order of their likelihood ratio. Tuscan red color indicates the cluster with the likelihood ratio and labeled cluster 1 (most likely cluster or primary cluster), while cluster 2 (flame red), cluster 3 (fire red), cluster 4 (mars red), cluster 5 (seville orange), and cluster 6 (mango) are secondary clusters from the highest to lowest likelihood ratio. Olive color indicates no cluster districts (Table 3)

The purely spatial cluster analysis result indicated a non-random distribution of under-five diarrhea incidence in Sidama Zone during July 2011–June 2017 (Table 3 and Fig. 5). Out of the 19 districts, eight of them (Boricha, Malga, Hulla, Aleta Wondo, Shebedino, Loka Abaya, Dale, and Wondogenet) had significantly higher cases than expected (log likelihood ratio greater than 1). Using the maximum spatial cluster size of ≤ 50% of the total population, one most likely cluster and six secondary clusters were identified. The most likely cluster had a relative risk (RR) of 1.82 (*p* < 0.001), with an observed number of cases of 29,153 and expected cases of 17,146.87. The RR of secondary clusters within a non-random distribution pattern was also significant (*p* < 0.001).

**Table 3:** Spatial clusters of under-five diarrhea in Southern Ethiopia between July 2011 and June 2017

Cluster number	District	Population	Coordinates	Obs.*	Exp^¥^	Annual cases/100,000	Obs./exp	RR	LLR	*P* value
Primary cluster	Boricha	49,076	6.939005 N, 38.253064 E	29,153	17,146.87	9898.2	1.70	1.82	3864.33	< 0.001
1st secondary cluster	Malga	21,530	6.933700 N, 38.562860 E	11,952	7522.60	9249.8	1.59	1.65	1154.94	< 0.001
2nd secondary cluster	Hulla	25,348	6.487117 N, 38.522366 E	13,335	8856.59	8765.7	1.51	1.54	1030.89	< 0.001
3rd secondary cluster	Shebedino	45,872	6.874400 N, 38.441810 E	19,636	16,027.43	7132.6	1.23	1.25	413.94	< 0.001
4th secondary cluster	Aleta Wondo	37,058	6.596820 N, 38.422840 E	16,005	12,947.92	7196.4	1.24	1.26	360.24	< 0.001
5th secondary cluster	Dale	47,585	6.745428 N, 38.409888 E)	17,713	16,626.00	6202.4	1.07	1.07	37.97	< 0.001
6th secondary cluster	Loka Abaya	19,459	6.694306 N, 38.202427 E	7351	6799.06	6294.4	1.08	1.08	22.60	< 0.001

*RR* relative risk, *LLR* log-likelihood ratio

*Number of observed cases in a cluster

^¥^Number of expected cases in a cluster

#### Purely temporal cluster

The purely temporal cluster analysis indicated that one most likely cluster was identified in all districts (LLR = 2109.93, *p* < 0.001) during December 2013 to May 2015 (01/12/2013 to 31/5/2015). The overall RR within the cluster was 1.37 (*p* < 0.001) with an observed number of cases of 63,683 and 50,695.23 expected cases. There was no secondary cluster identified.

#### Spatiotemporal clusters of childhood diarrhea

The space-time cluster analysis of cases of under-five diarrhea from July 2011 to June 2017 in Sidama Zone showed that diarrhea was not distributed randomly in space-time. Using the maximum spatial cluster size of 50% of the total population, and the maximum temporal cluster size of 50% of the total population, one most likely cluster and four secondary clusters were identified (Table 4). The overall RR within the most likely cluster was 2.03 (*p* < 0.001) with an observed number of cases of 6186 compared with expected cases of 3097.21. The RR of secondary clusters, within a non-random distribution pattern, was also significant (*p* < 0.001).

**Table 4:** Spatiotemporal clusters of under-five diarrhea in Southern Ethiopia between July 2011 and June 2017

Cluster number	District	Population	Coordinates	Time frame	Obs.*	Exp^¥^	Annual cases/100,000	Observed/expected	RR	LLR	*P* value
1	Malga	21,530	6.933700 N, 38.562860 E	2012/12/1 to 2015/5/31	6186	3097.21	11,627.8	2.00	2.03	1214.67	< 0.001
2	Loka Abaya	19,459	6.694306 N, 38.202427 E	2012/1/1 to 2012/5/31	900	441.07	11,879.4	2.04	2.05	183.47	< 0.001
3	Bursa	20,322	6.590160 N, 38.606330 E	2011/12/1 to 2011/12/31	327	93.27	20,410.6	3.51	3.51	176.61	< 0.001
4	Gorchie	20,683	6.876740 N, 38.584650 E	2014/3/1 to 2014/4/30	479	199.43	13,983.0	2.40	2.41	140.34	< 0.001
5	Wensho	17,583	6.749010 N, 38.517450 E	2012/3/1 to 2012/3/31	216	81.28	15,471.9	2.66	2.66	76.44	< 0.001

*RR* relative risk, *LLR* log-likelihood ratio

*Number of observed cases in a cluster

^¥^Number of expected cases in a cluster

## Discussion

In this study, seasonal variation and hotspots of under-five diarrhea are indicated. The 6-year monthly under-five diarrhea report shows an overall increasing trend and seasonal variation in the study area. The highest incidence rate peaked in Boricha district in the year 2016/17. Spatial, temporal, and space-time hotspots of diarrhea were also observed in Boricha, Malga, Hulla, Loka Abaya, Bursa, Gorchie, and Wonsho districts of Southern Ethiopia.

The current study showed that childhood diarrhea occurred in a cyclical pattern over the months of the 6 years study period. Previous reports found this. For example, in Brazil, hospitalization rate caused by acute diarrhea in children under the age of one showed annual seasonal and 6-monthly patterns [20]. A study in Northwest Ethiopia also showed that peak childhood diarrheal cases showed a seasonal trend [14]. In China, diarrhea in children under 5 years showed a bimodal distribution, where it showed its peak in fall-winter seasons [43].

Despite reported improvements in water, sanitation, hygiene, and vaccination coverage in the study area over the years, the annual number of reported childhood diarrheal cases showed an increasing trend. Our finding contradicts the previous report in Gojam, Northwest Ethiopia, where childhood diarrhea showed a decreasing trend [14]. The previous four DHS reports of Ethiopia also showed a decreasing trend in childhood diarrhea in [44]. Another study based on DHS data in Burkina Faso, Mali, Nigeria, and Niger during the period between 1990 and 2013 identified a decrease in the proportions of diarrheal morbidity among under-five children [45]. The fact that the childhood diarrhea morbidity showed an increasing trend over the years might be because newly built health facilities started reporting the diarrheal morbidity and health extension workers, who previously did not diagnose and treat diarrhea, have started diagnosing, treating, and reporting of diarrhea morbidity. Our data suggest an annual diarrheal incidence of around six cases per 100 while the DHS data implies an annual incidence order of magnitude higher, suggesting that the great majority of cases are not reported in the HMIS. This is because the DHS study is based on a community-based survey, whereas the current study is based on the report of cases who visited health institutions seeking medical assistance.

The 6 years childhood diarrhea incidence starts to increase in January and reaches its peak in February. According to the historical (1983–1984) monthly data of rainfall, maximum temperature, and minimum temperature, this is the transition from driest to the rainy season. During this time, the average maximum temperature reaches its highest [39]. Similar studies also showed that increase in temperature was positively associated with diarrhea incidence [46, 47]. The incidence rate starts to slowly decline through time to reach its lowest peaks in the months of July to November. Shortage of water in the dry season has been associated with increased prevalence of diarrhea [22]. This may be due to less availability of fresh water or concentration of contaminants in smaller volumes of water [48] or longer water storage [49]. A large outbreak of diarrhea occurred following severe droughts due to decreased water availability and worsened personal hygiene [50]. Extreme rainfall days and associated flooding were also strongly related to diarrhea-associated morbidity [51, 52]. This is because flooding could result in the breakdown of sanitary conditions and contamination of drinking water sources by washing nutrients, pathogens, and toxins into water bodies [53].

The study also showed the existence of substantial variation in the spatial distribution of diarrhea within the study area. The finding was in agreement with another national study [15]. However, there is the difference in the nature of the data sources between the studies in that the later used cross-sectional DHS data. The highest risk of diarrhea was found in Boricha district. This might be due to the fact that most of the residents of the district relied heavily on pond water, which is open for both human and animal. In addition, the population is agro-pastoralist, where they move from place to place in search of food and water for their animals. These types of people would not have the chance to construct and use their own latrines. It can also be due to other factors such as differing levels of poverty, education, and lifestyle.

Spatial hotspots of diarrhea were also observed in Malga and Hulla districts. This might be because these districts are located in Highlands, where most people share their home with their domestic animals at night because of fear of cold and theft. It is estimated that around 90% of rural households in Ethiopia own some farm animals [54]. Sharing of the dwellings with livestock is quite common [55, 56]. This has been linked to disease [55, 57]. One of the main routes of transmission of diarrheic agents is through domesticated animals as they serve as reservoirs for various zoonotic diseases agents and also to other domestic and wild animals [58–61]. The presence of domestic animals around the dwellings can compromise the sanitation of the household and their neighborhood environment, and thus, it increases the chance of the dwellers come in contact with animal droppings; thereby, there are chances of vertical transmission of the microbes to the owners [62, 63]. Zoonotic diseases such as *Campylobacter* diarrhea, *Cryptosporidium* diarrhea, and *E. coli* O157 infection have been reported following exposure to unhygienic environments as a result of domestic animals living in and around the human dwellings [61, 64, 65].

The findings showed temporal variation in the overall risk of diarrhea, which indicated that childhood diarrhea was not distributed randomly in time. This might be due to the influence of socio-economical, environmental, or climate-related factors. Statistically significant space-time hotspots (*p* < 0.001) were also observed (Table 4). This consisted of a primary hotspot and four secondary hotspots. The primary hotspot was observed in December 2012 to January 2015 in Malga district with a likelihood ratio of 1214.67 and relative risk of 2.03. The first, second, third, and fourth secondary hotspots occurred from January 2012 to May 2012 in Loka Abaya, December 2011 in Bursa, from March to April 2014 in Gorchie, and March 2012 in Wonsho districts. The primary hotspot spanned for slightly more than 2 years, and the other secondary clusters existed from 1 to 5 months only. This might be due to the occurrence of risk factors specific to the local areas and time periods.

This study has strengths and limitations. The fact that complete monthly morbidity data were obtained from all districts throughout the study period was a strength. The other strength was the existence of historical 30 years precipitation, maximum temperature, and minimum temperature data which were compared with the seasonal variation of childhood diarrhea that could have been influenced by metrological parameters. The use of the SaTScan software allowed us to both detect the location of clusters and evaluate their statistical significance without problems with multiple testing. A limitation is under-reporting of childhood diarrhea as the data reflects only those who sought healthcare setup level treatment. As a result, the study might not be indicative of the true picture of diarrheal morbidity of the study area. However, this limitation might be minimal as the problem is assumed to be uniform across all districts over the study period.

## Conclusions

This study assessed the seasonal variation and spatial, temporal, and space-time clusters of under-five diarrhea using 6 years HMIS morbidity data (July 2011 to June 2017) in Southern Ethiopia. An increasing trend with a seasonal variation of childhood diarrhea which peaks in the transition period from driest to the rainy season occurred. An excess risk of diarrhea was also observed in Boricha, Malga, and Hulla districts. Statistically significant space-time hotspots were also observed in five districts from December 2012 to January 2015 in Malga district, from January 2012 to May 2012 in Loka Abaya, December 2011 in Bursa, from March to April 2014 in Gorchie, and March 2012 in Wonsho districts.

The spatial, temporal, and space-time clusters, generated in this research, can be used by the various stakeholders to prioritize places of intervention. In addition, season-specific interventional strategies can be developed with efficient resource use to reduce the childhood morbidity, mortality, and financial losses related to visiting health instructions as a result of diarrhea morbidity. Further studies are required to clarify the effect of weather variabilities on under-five diarrhea incidence and to investigate the specific risk factors of childhood diarrhea in hotspot areas.

## Additional file

Additional file 1: Time series plot of under-five diarrhea incidence rate per 1000. (XLSX 31 kb)
